# Supplementary material for: A Morphological and Morphometric Dental Analysis as a Forensic Tool to Identify the Iberian Wolf (Canis Lupus Signatus)
Source: Animals (Basel). 2020 Jun 3;10(6):975. doi: 10.3390/ani10060975 (PMC7341320; doi:10.3390/ani10060975)
Supplement: Supplementary file 1 [file animals-10-00975-s001.pdf]

Supplementary Materials: A Morphological and Morphometric Dental Analysis as a Forensic Tool to Identify the Iberian Wolf (*Canis Lupus Signatus*)

Víctor Toledo González, Fernando Ortega Ojeda, Gabriel M. Fonseca, Carmen García-Ruiz, Pablo Navarro Cáceres, Pilar Pérez-Lloret and María del Pilar Marín García

Table S1. Comparison of the mean values for the wolf sex differentiation (95% confidence) of 26 female and 19 male skulls. The comparison considered the existence or exclusion of the outlier values present only in seven specific variables. Va, variable; F, females; M, males; mm, millimetres.

Va		Mean with the outlier values (mm)	Mean without the outlier values (mm)
UiW	F	32.04	32.04
	M	34.35	34.18
L1Mc	F	48.42	50.54
	M	52.52	54.28
UrCWd	F	8.12	8.28
	M	9.23	9.23
UlCLe	F	12.62	12.82
	M	13.99	13.99
LrCLe	F	12.95	12.70
	M	14.27	14.27
LlCLe	F	12.76	12.48
	M	14.03	14.02
Ur4PMtub	F	16.36	16.41
	M	17.02	17.02

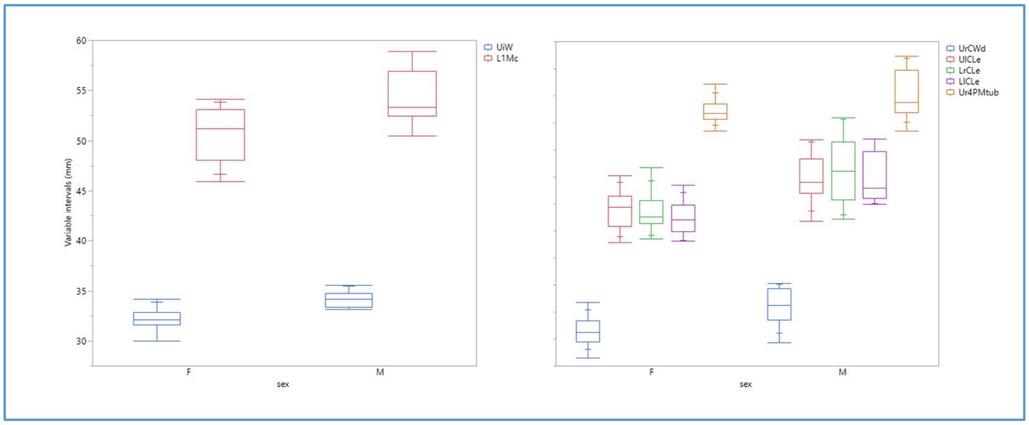

Figure S1. Boxplots for the dental measurements without the outlier values considering sex as a source of variation. The intervals include the minimum, maximum, mean, and quartiles values for each variable according to sex.

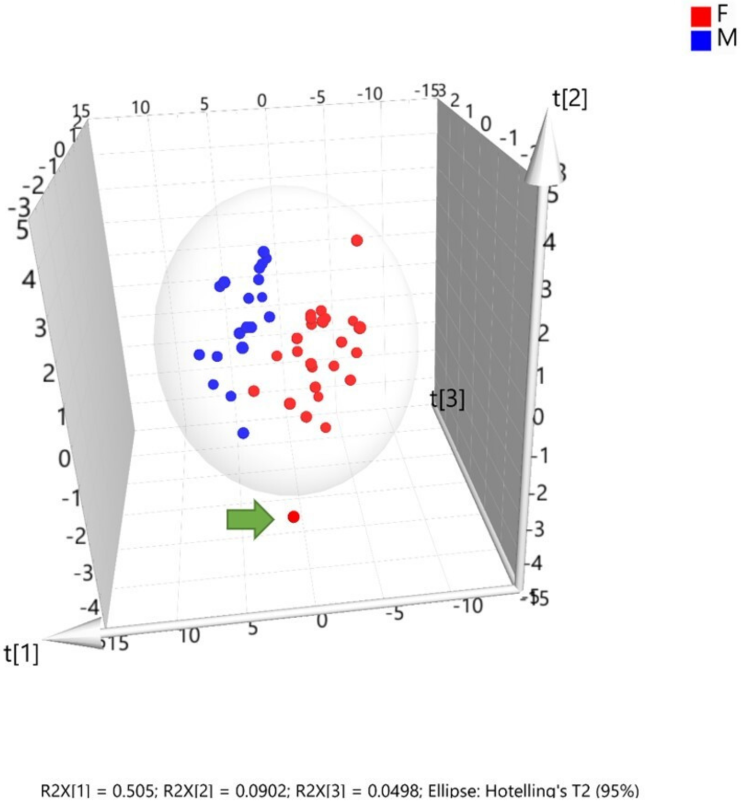

Figure S2. The 3D scatter PLS-DA plot showing the sex differentiation before the outlier female sample (green arrow) was excluded from the multivariate analysis. The outlier sample was outside the Hotelling's T2 95 % confidence level. The initial latent variables t[1], t[2], and t[3], explained 50.5, 9.02, and 4.98% of the variability, respectively. The whole model explained 64.5 % of the variance.
